# Novel Conformal Skin Patch with Embedded Thin-Film Electrodes for Early Detection of Extravasation

**DOI:** 10.3390/s21103429

**Published:** 2021-05-14

**Authors:** Ruiqi Lim, Ramona B. Damalerio, Choon Looi Bong, Swee Kim Tan, Ming-Yuan Cheng

**Affiliations:** 1 Institute of Microelectronics, A*STAR (Agency for Science, Technology and Research), Singapore 138634, Singapore; limrq@ime.a-star.edu.sg (R.L.); damaleriom@ime.a-star.edu.sg (R.B.D.); 2 KK Women’s & Children’s Hospital, Singapore 229899, Singapore; bong.choon.looi@singhealth.com.sg (C.L.B.); josephine.tan.s.k@singhealth.com.sg (S.K.T.)

**Keywords:** extravasations, wearable, sensor, medical, noninvasive

## Abstract

Extravasation is a complication of intravenous (IV) cannulation in which vesicant drugs leak from a vein into the surrounding subcutaneous tissue. The severity of extravasation depends on the type, concentration, and volume of drugs that accumulate in the subcutaneous tissue. Rapid detection of extravasation can facilitate prompt medical intervention, minimizing tissue damage, and preventing adverse events. In this study, we present two portable sensor patches, namely gold- and carbon-based sensing patches, for early detection of extravasation. The gold-based sensor patch detected extravasated fluid of volume as low as 2 mL in in vivo animal models and human clinical trials; the patch exhibited a resistance change of 41%. The carbon-based sensor patch exhibited a resistance change of 51% for 2 mL of extravasated fluid, and fabrication throughput and cost-effectiveness are superior for this patch compared with the gold-based sensing patch.

## 1. Introduction

**Figure 1:**
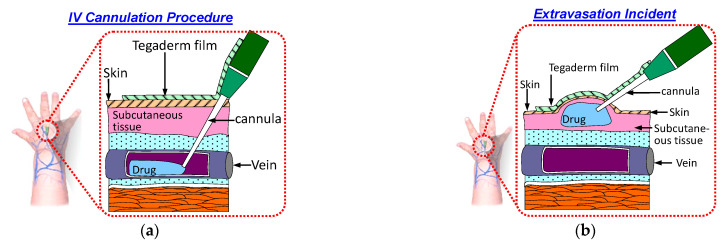
Schematic of the IV cannulation procedure: (**a**) successful IV cannulation; (**b**) extravasation incident.

Intravenous (IV) cannulation is a common procedure for drug and fluid administration. In this procedure, an IV cannula is inserted into a patient’s vein to enable drug and fluid administration as well as blood sampling. The cannula must remain in the patient’s vein throughout the IV infusion. If the cannula is displaced from the patient’s vein during the infusion, then the medication will be infused into the surrounding tissue. This phenomenon is called extravasation and results in complications. Figure 1a displays a schematic of a typical IV cannulation procedure. An adhesive Tegaderm film is used to fix the cannula to prevent it being dislodged from the vein.

Extravasation injury refers to the damage resulting from vesicant medication leakage into surrounding tissue. This results in swelling of the skin around the cannulation site, as illustrated in Figure 1b. Various factors contribute to the occurrence of extravasation. One factor is the small size and fragile nature of veins in infants and older patients, respectively. Another group of patients at risk of extravasation are those undergoing chemotherapy; these patients have compromised veins due to frequent venipunctures. Another factor is the movement of patients during IV infusion. This movement can lead to accidental displacement of the cannula from the vein [1]. The incidence of extravasation in patients undergoing chemotherapy and newborns is approximately 0.1–0.6% [2] and 11% [3], respectively.

The severity of extravasation injuries depends on the type, concentration, and volume of the drug that was extravasated into the tissue layer. Vesicant medication refers to solutions with a pH of <5.5 or >8.5 or solutions with osmolarity > 290 mOsmol/L [4,5,6]. Vesicant drugs result in severe tissue damage and adverse events because these drugs possess DNA binding ability; they can also kill replication cells that come into contact with them or cause vascular dilation. The volume of extravasated drug fluid in the tissue space depends on the infusion rate and the time that elapses from extravasation occurrence to detection by nursing staff [7]. The severity of extravasation injury can vary from mild effects (e.g., redness, pain, and blistering) to critical conditions such as tissue necrosis, which requires surgical intervention such as debridement, skin grafting, or amputation. Patients with impaired levels of consciousness, neonates, and young children cannot alert nursing staff if they experience pain from extravasation. Therefore, extravasation detection is often delayed, which can result in severe injuries because of the continuous accumulation of fluid in subcutaneous tissue. Approximately 4% of neonates with extravasation injuries develop cosmetic or functional scars [7] and 0.38% develop tissue necrosis [8]. Therefore, early extravasation detection is critical for facilitating prompt medical intervention to minimize tissue damage.

Changes in various tissue parameters, such as tissue permittivity, permeability, temperature, impedance, blood flow rate, and blister formation [9,10,11,12,13,14,15,16,17], are used as extravasation indicators. Current extravasation detection tools, such as the MEDRAD extravasation detection system (MEDRAD XDS) [9,10] and extravasation detection accessory (EDA) systems [11,12,13], are mainly employed in computed tomography (CT). In the MEDRAD XDS, radio frequency waves are used for the detection of fluid accumulation under patients’ skin. Fluid pooling of volume less than 20 mL can be detected during CT. An EDA extravasation detection system, comprising an electrode patch with an embedded sensor, is included in the Empower CTA+ Injector System. The EDA system measures the change in skin impedance as an indicator of extravasation. The injection of fluid is paused by EDA systems if a clinically significant variation is detected. However, EDA systems are useful only at high flow rates of >2.5 mL/s, with an average detectable volume of 12.5 mL in the subcutaneous tissue. Both MEDRAD XDS and EDA equipment can detect large volumes (>12.5 mL) of extravasation but are not sufficiently sensitive to detect extravasation at an early stage. Moreover, both MEDRAD XDS and EDA systems are bulky, expensive, and unsuitable for routine use for numerous patients. Thus, the development of a portable and cost-effective device for detecting extravasation during routine IV cannulation is critical.

Bicen et al. presented various sensing modalities for extravasation applications given an infusion flow rate of 2 mL/min [9]. The bioresistance and bioreactance modality exhibited low detection probability of approximately 0.5 and 0.66 for infusion volumes 2 and 5 mL, respectively. The detection probability was higher for infusion volumes >10 mL. The detection probability of the skin strain sensing modality was >0.9 and 0.87 for infusion volumes 2 and 5 mL, respectively.

The current study proposes piezoresistive sensor patches for early detection of extravasation for as little as 2 mL of infused fluid and with a minimum resistance change of 40%. The sensor patch is attached to the cannula site, and its resistance changes if a bump forms under the skin. This procedure aids early detection of extravasation and minimizes the severity of tissue injury. Sensor design details as well as testing results of ex vivo, in vivo, and clinical trial data are presented in the following sections.

## 2. Design Concept

**Figure 2:**
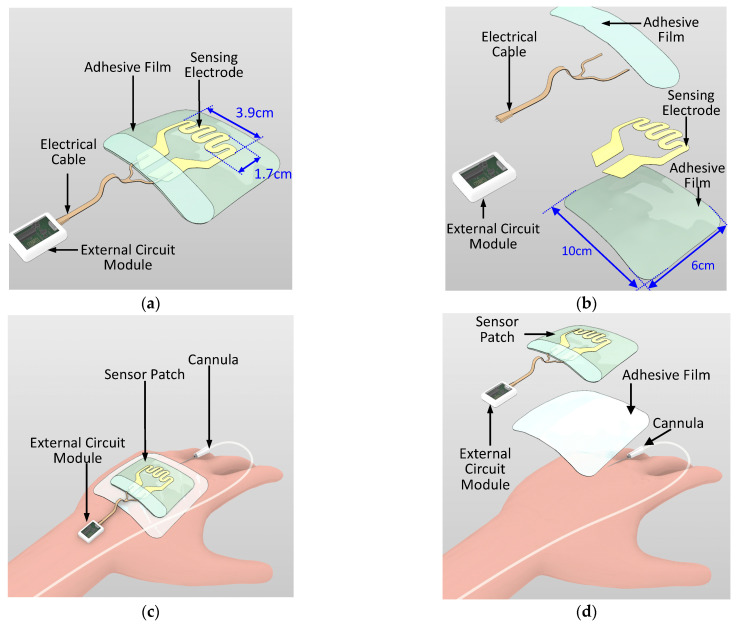
Schematic of the sensor patch system: (**a**) overview, (**b**) exploded view, (**c**) sensor patch system on a patient’s hand with the cannula, and (**d**) exploded view of the sensor patch system on the patient’s hand.

Wearable sensor development for various medical and wellness applications is a growing trend. Examples of such sensors include skin patches for epidermal vital sign monitoring systems and wound monitoring [18,19,20,21,22,23,24]. In the current study, a novel sensor patch system is proposed for the early detection of extravasation. The sensor patch system consists of two main components, namely a disposable sensor patch and a reusable external circuit reader (Figure 2a,b). In the disposable sensor patch, 3M Tegaderm film (length: 10 cm; width: 6 cm) is used as the base adhesive substrate and a piezoresistive sensing electrode is deposited over this substrate. The sensing electrode has a serpentine pattern with sensing area 3.9 cm × 1.7 cm (Figure 2a). The position of the sensing electrode is such that it covers the region surrounding the tip of the cannula where skin swelling due to extravasation is most likely to occur. Compared with a simple linear sensor shape design that is one-dimensional, the two-dimensional serpentine design covers a larger sensing region and results in higher detection sensitivity. The sensing electrode is patterned using two piezoresistive sensitive materials, namely gold and carbon conductive inks. The sensing electrode is then electrically connected through a conductive cable to the external circuit module. As per standard IV cannulation procedure, a Tegaderm film is directly placed over the cannula to secure the cannula. The sensor patch is placed over the Tegaderm film (Figure 2c,d). The sensing patch has wide detection coverage over the hand of the patient and the sensing patch does not come into direct contact with the skin surface. Thus, no additional IV cannulation steps are required for the nursing staff when applying this sensor patch.

When extravasation occurs, the accumulation of drug in subcutaneous tissue results in swelling under the skin. The sensor patch on the skin surface is then stretched, as illustrated in Figure 1b. This stretching results in sensing element deformation and changes the sensing electrode’s resistance. An external circuit module can be used to record this sudden change in the sensing electrode’s resistance and trigger an alert to the nursing staff for their immediate intervention. A member of the nursing staff can then inspect the cannula site and intervene. Therefore, early extravasation detection is critical for management of extravasation injury and prevention of adverse tissue damage.

## 3. Fabrication and Microassembly

**Figure 3:**
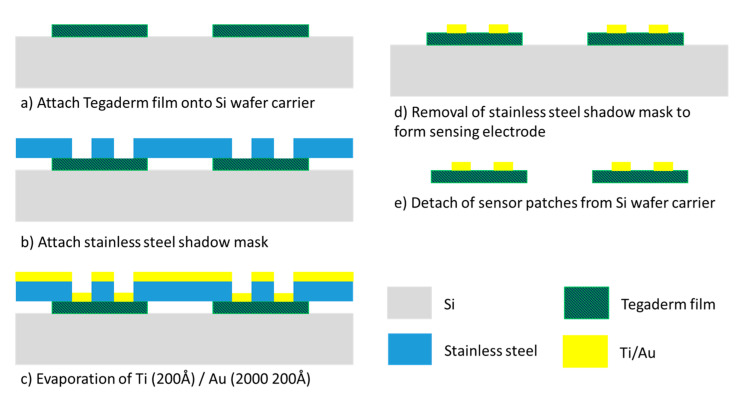
Gold-based sensing patch process flow.

The fabrication of the gold-based sensing patch involved a one-layer semiconductor process (Figure 3). A stainless-steel stencil with sensor electrode pattern was fabricated through laser machining. This stencil functioned as the shadow mask for the gold metal evaporation process. Tegaderm films functioned as the base substrate for sensor patches and were attached to an 8-inch silicon carrier wafer followed by the stencil mask. A thin 200 Å titanium layer was first evaporated onto the Tegaderm film to act as an adhesion layer for the gold layer. Subsequently, a 2000 Å gold layer was evaporated on top of the titanium layer. The shadow mask was then detached from the Tegaderm films to form the sensing electrode pattern. A disadvantage of this process is the manufacturing cost. Because of the limited chamber size of the evaporator machine, the machine was able to process only approximately 40 sensor patches per run. This method results in considerable cost of manufacturing the sensor patch and means that gold is a relatively unattractive material for the sensing electrode.

**Figure 4:**
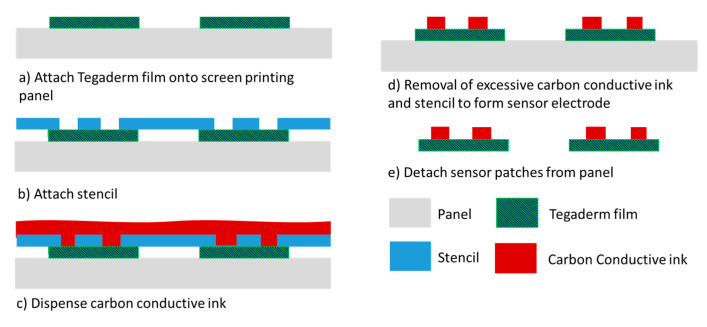
Carbon conductive ink–based sensing patch process flow.

To address the high cost of fabricating gold-based sensor patches, patches of an alternative cost-effective piezoresistive material, carbon conductive ink, were also investigated. The fabrication of the carbon-based sensor patches involved screen printing (Figure 4), which is a panel-level process in which multiple Tegaderm films can be aligned and attached to the panel substrate in each process run. Approximately 100 sensor patches can be processed per process run. Furthermore, screen printing is faster than the evaporation process. The greater throughput and shorter processing time result in considerably more cost-effective sensor patch fabrication.

**Figure 5:**
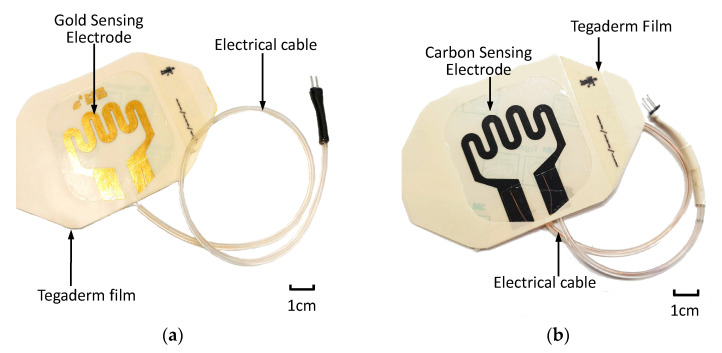
Assembled extravasation sensor patch: (**a**) gold-based and (**b**) carbon-based sensing patches.

After sensing electrode patterning was conducted using commercially available carbon conductive ink (MOS Corporation, China), electrical cables were formed using copper wires, insulating tubing and a two-pin header. Electrical cables were assembled onto the bonding pads of the sensor patch. An additional adhesive film was used to secure the electrical cable to the sensor patch. Figure 5 displays the assembled sensor patches with the gold-based and carbon conductive ink–based sensor electrodes.

## 4. Results and Discussion

### 4.1. In Vivo Testing of Gold-Based Sensing Patch

**Figure 6:**
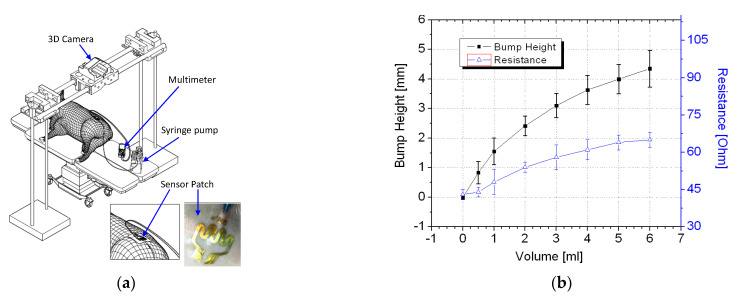
In vivo experiment: (**a**) schematic of the in vivo trial setup and (**b**) test results.

In vivo trials were conducted using a piglet model. Regulatory approval for the animal study was obtained from the relevant institutional animal care and use committee (reference 2014/SHS/907). Figure 6a illustrates the in vivo trial setup. The piglet was first sedated before a syringe pump was used to inject up to 6 mL of a saline solution into the piglet. For application of the sensor patch to extravasation detection, the ability of a sensor patch to detect less than 5 mL of infused volume in human is considered a substantial improvement over existing methods [20,21,22,23,24].

Thus, volumes of up to 6 mL of saline solution were infused into the animal models for validation of the sensor patches’ performance. At each measured infused volume, a bump formed and the sensor patch was stretched. This changed its resistance, which was measured using a digital multimeter.

**Figure 7:**
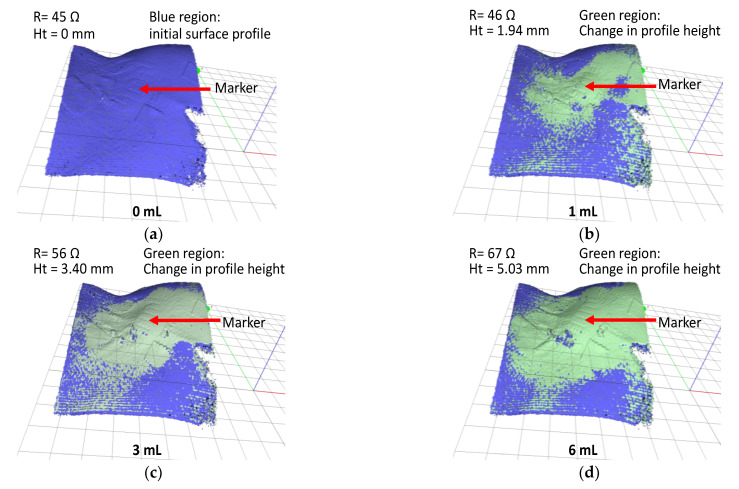
In vivo test result; 3D scan images of extravasation bump on a pig hind limb for fluid infusion volumes (**a**) 0, (**b**) 1, (**c**) 3, and (**d**) 6 mL.

The piglet was tested three times using different sensor patches. The average resistance data with two standard deviations are displayed in Figure 6b. The results of the trials confirmed that the tissue swelling caused by extravasation can be detected using the sensor patch. No false-negative result were detected during the trial. The sensor patch also exhibited high sensitivity at ≤2 mL infused volume for early detection of extravasation. A three-dimensional (3D) scanner (Artec 3D, Luxembourg) was positioned directly above the cannulation site to scan the surface structure for each measured infused volume. To facilitate the measurement of bump height, multiple types of polymer-based tape were placed on the sensor patch. The polymer tape acted as a marker during the 3D scanning procedure. After the polymer tape had been placed, an initialization procedure was conducted at 0 mL infusion, and the surface profile height of the marker was set as the baseline height (0 mm). Subsequently, a 3D scan was conducted at each measured infused volume. The 3D scans were postprocessed to measure the change in extravasation bump formation with reference to the baseline measurement, specifically the bump height (Figure 7).

First, a 3D scan was performed prior to the start of the experiment (infusion volume = 0 mL) to generate the baseline surface profile of the animal model (blue region in Figure 7a). Subsequently, a sequence of 3D scans was conducted at 1 mL of infusion volumes interval. The changes in surface profile height are indicated by the green regions in Figure 7b–d. At an infusion volume of 6 mL, the change in the surface profile height was 5.03 mm.

### 4.2. Clinical Trial for Gold-Based Sensing Patch

**Figure 8:**
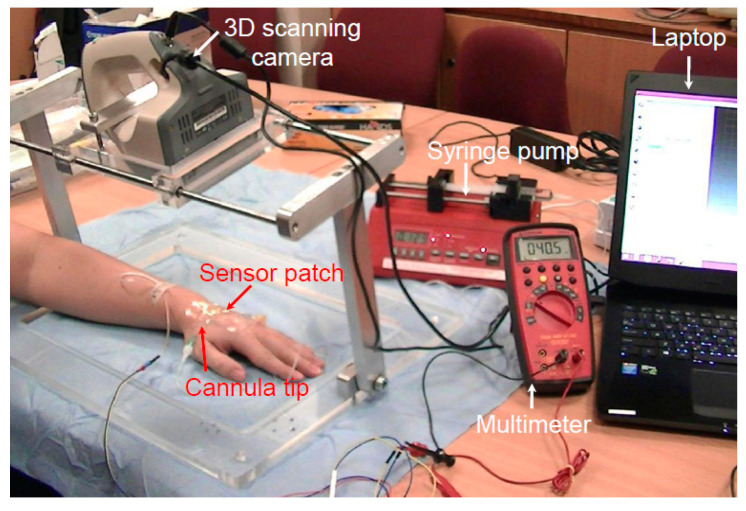
Setup of the human clinical trial.

The tests performed on live pig models revealed that the device is safe and effective when used to detect the early stage of extravasation. To determine whether the device is similarly effective in humans, a clinical trial was conducted to simulate extravasation conditions. Regulatory approval from SingHealth Centralised Institutional Review Board was obtained for a clinical study involving nine healthy volunteers (reference 2015/2452). Table 1 presents details of the volunteers. Forty-five minutes prior to cannulation, a topical local anesthetic cream (Ametop) was applied to the dorsum of the volunteers’ nondominant hand to anesthetize the skin. A 22-gauge cannula was placed under the skin in the subcutaneous tissue, deliberately avoiding the vein. Figure 8 displays the setup of the clinical study. Cannulation was performed by a skilled pediatric anesthetist. A standard adhesive film dressing was used to secure the cannula, and the extravasation sensor patch system was placed over it. After the system had been set up, each participant was subjected to one cycle of normal saline infusion of up to 2 mL into the subcutaneous tissue at a rate of 50 mL/h to simulate extravasation bump formation.

Various devices for early detection of extravasation have been reported [9,11,25]. These devices can detect 2–10 mL of extravasated volume for early detection to minimize adverse tissue damage. The proposed sensor patch aims to detect extravasation as early as possible. Hence, an infusion volume of 2 mL and a minimum of a 40% change in resistance were selected as the benchmarking criteria for sensor patch validation.

**Figure 9:**
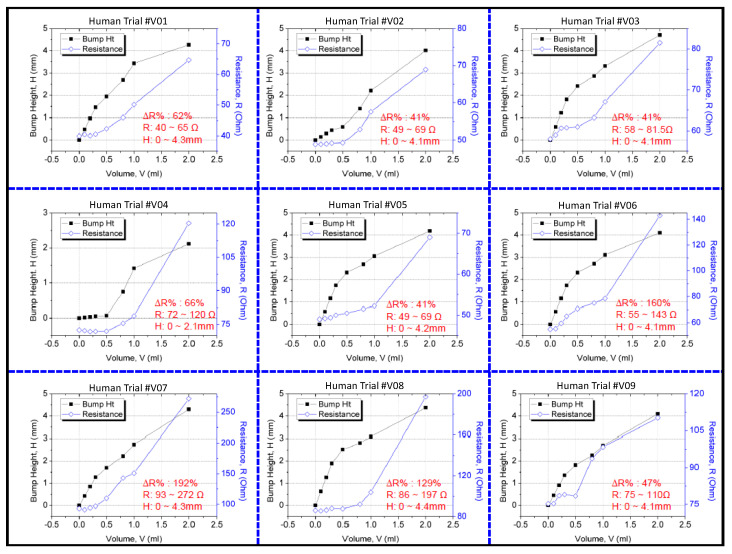
Results of the clinical study on the sensor patch.

**Figure 10:**
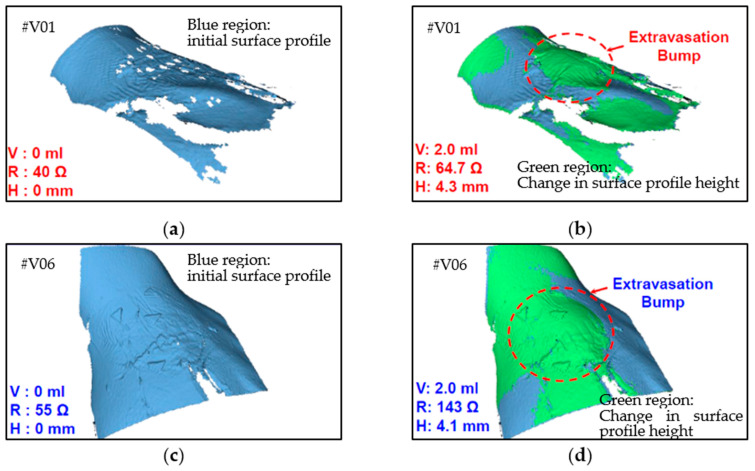
Three-dimensional images of two human volunteers with extravasation injury: (**a**) V01 at 0 mL infusion, (**b**) V01 at 2 mL infusion, (**c**) V06 at 0 mL infusion, and (**d**) V06 at 2 mL infusion.

The resistance change was measured using a multimeter, and the 3D bump formation was recorded using the digital 3D scanner. Figure 9 displays the results of the clinical trial. Because people differ in term of skin properties, fluid infusion and accumulation into the subcutaneous tissue may vary. Thus, the differences in bump height and resistance that were observed between the participants were expected. However, the sensing electrode’s resistance was discovered to increase exponentially and to increase to higher than the 40% minimum threshold for 2 mL of infused volume in the trials. This result verified that the device could effectively detect extravasation at an early stage in humans at an infusion volume of 2 mL. No false-negative results were detected in the nine participants. The 3D scanner images were used to detect the site of maximal fluid accumulation following extravasation (Figure 10). The blue regions in the scan images of the hand denote the initial hand surface profile. The change in surface profile is denoted by the green regions in Figure 10. The bump height ranged between 2.1 and 4.4 mm for 2 mL of infused volume. These bumps are not clear under visual inspection and would thus probably be undetected by nursing staff.

**Figure 11:**
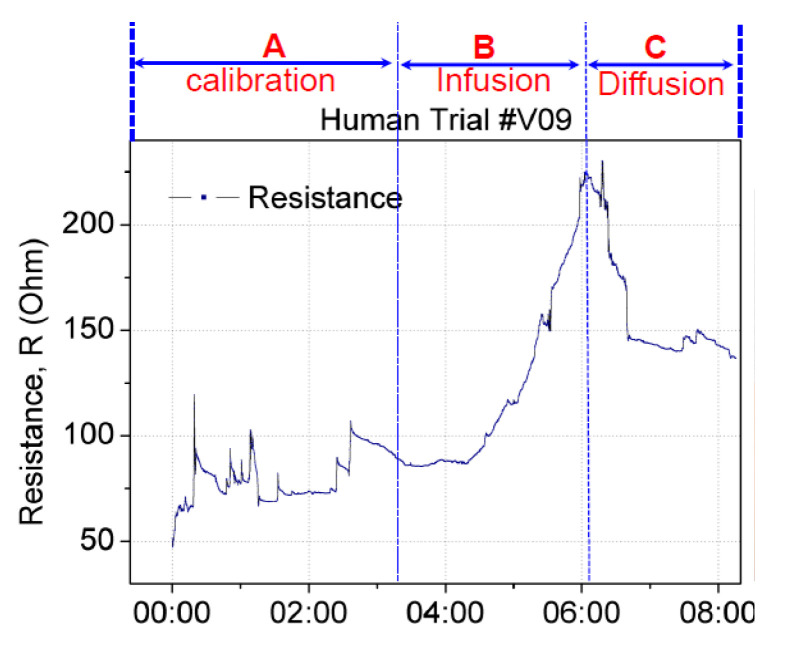
Real-time changes in the resistance of the sensor patch under extravasation. Calibration was performed by rotating the hand with cannulation by ±15° to the left and right before fluid infusion.

False positives can occur when a sensor patch identifies extravasation even though the cannula remains in the vein. A false positive scenario may arise because of the movement of the patient’s hand or unintentional stretching of the sensor patch. To validate the sensor patch for false positive scenarios, two test studies were conducted. In the first study, the sensor patch was tested under hand movement. Using the same setup as that described previously, the hand with the cannulation site was moved or rotated by ±15° to the left and right. This movement was set as the baseline calibration before infusion and diffusion. Figure 11 displays the change in the resistance in real time. Section A denotes the baseline calibration in which saline infusion was performed from 0:00 to 3:20 min, with a resistance change from 50 to 100 Ω. Section B denotes saline infusion from 3:21 to 6:04 min, with a resistance change from 85 to 220 Ω. The diffusion in Section C started from 6:05 and continued until 8:05 min, with a resistance change from 220 to 140 Ω. This setup confirmed that the resistance increased with infusion and decreased with diffusion or changed with respect to the stretching of the sensor patch. The amplitude and trend of resistance change caused by hand-movement-induced fluctuation in Section A differed from those for extravasation. The resistance increase resulting from extravasation is exponential. Thus, algorithm can be used to filter out the hand-movement-induced fluctuation signal from extravasation signal.

**Figure 12:**
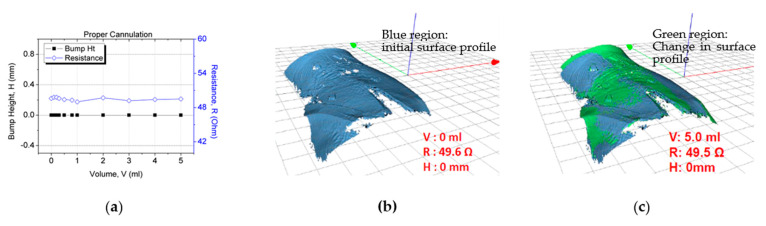
Sensor patch after proper cannulation: (**a**) test result, (**b**) 3D image after proper cannulation (0 mL), and (**c**) no bump formation after 5 mL fluid infusion.

In the second study for sensor patch validation in false positive scenarios, negative control testing was conducted in which the participants underwent the proper cannulation procedure. The objective of the experiment was to verify that the resistance reading does not vary with an increase in the fluid infusion volume. The same trial setup and skin preparation as described previously were used for this test. The cannula was inserted into the vein instead of subcutaneous tissue for these tests, observing correct cannulation procedures. When the system was set up, 5 mL of normal saline was injected through the cannula at a rate of 50 mL/h. After 5 mL of fluid had been infused, the resistance value (~49.6 Ω) had not changed (Figure 12a). Furthermore, no extravasation bump formed (height: ~0 mm; Figure 12). This result validated the sensor patch’s functionality under proper cannulation with no false positives or notable changes in the sensor electrode resistance.

### 4.3. Ex Vivo Testing of the Carbon-Based Sensing Patch

**Figure 13:**
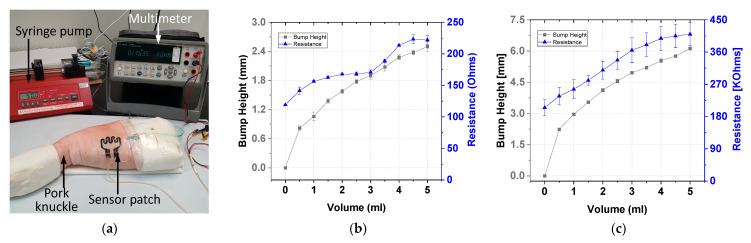
Ex vivo testing: (**a**) experiment setup and test results for the (**b**) gold-based and (**c**) carbon-based sensing patches.

Ex vivo tests were conducted using carbon-based sensor electrodes for comparing the gold-based and carbon-based sensing patches. Figure 13a displays the ex vivo test setup of the sensor in which the sensor patch was secured over the cannula site on pork knuckle. To simulate extravasation of fluids in subcutaneous tissue, 5 mL of contrast solution was infused into the tissue at a rate of 50 mL/h. This accumulated fluid caused a bump in the pork knuckle. The pork knuckle surface topology was scanned, and the bump height was measured and recorded using a 3D camera at 1-mL infusion volume intervals. The change in the sensing electrode’s resistance was measured using a multimeter. Three sets of data per sensor type were collected. The average resistance and bump height result with two standard deviations are displayed in Figure 13. The gold-based sensing patch exhibited an average 41% increase in resistance, whereas the carbon-based sensing patch exhibited an average resistance change of 51%. The performance of the carbon-based sensing patch was comparable with that of the gold-based sensing patch in terms of resistance change at 2 mL infused volume.

The carbon-based sensing patch exhibited resistance with greater variance than the gold-based sensing patch. This was due to the different methods used for fabricating the two sensing patches. The gold-based sensing patch was fabricated using a semiconductor evaporation process, whereas the carbon-based sensing patch was fabricated using screen printing. The gold evaporation process is more uniform and stable than the screen printing technique. Thus, compared with the carbon-based patch, the gold-based sensing patch has lower variance (Figure 13). However, carbon-based sensing patches have higher manufacturing throughput because of the use of the screen printing technique. Thus, the carbon-based sensing patch is more cost-effective and beneficial to end users than is the gold-based sensing patch.

## 5. Conclusions

This study presented gold- and carbon-based sensing patches. These sensor patches can be applied to patients to prevent extravasation injuries. The sensor patches can be attached to the skin in a similar manner as an adhesive film dressing to secure the cannula as part of the standard IV cannulation procedure. The performance of the sensor patches was validated through in vivo animal models and a clinical trial.

Animal trial was conducted on a piglet model to validate the performance of the sensor patches and evaluate the effect of false negative scenarios. The developed patches detected the skin swelling caused by extravasation. No false negative results were detected for 2 mL infusion volume. The gold-based sensing patch exhibited high sensitivity for extravasation detection at 2 mL fluid infusion volume.

Human trials were successfully conducted with nine volunteers and using the gold-based sensing patch. Extravasation injury conditions were simulated using cannulation in the subcutaneous tissue and infusion of 2 mL of normal saline solution at an infusion rate of 50 mL/h. The size of the extravasation bump was 0–4.2 mm, and the measured resistance change was 41–197%. The criterion for early detection was defined as a minimum 40% change in the sensing electrode’s resistance at 2 mL infused solution. The current results revealed that the sensor patch could detect extravasation at 2 mL infused volume for all participants. No false negative results were detected for an infusion volume of 2 mL. The gold-based sensor patch was validated using a negative control test condition and normal IV cannulation. On injection of 5 mL of normal saline into the volunteers’ veins, no change was observed in the bump height (H = 0 mm), and the measured resistance change was negligible (0.01%). A third testing condition was used to validate the resistance change caused by hand movement. The result revealed that the resistance change fluctuation due to movement differed from that due to extravasation. Thus, false positives caused by hand movements can be averted. The human trial demonstrated the functionality and ability of the gold-based sensing patch in early detection of extravasation for fluid volume of 2 mL in subcutaneous tissue.

The developed carbon-based sensing patch can address the high cost and low-throughput fabrication concerns faced with gold-based sensing patches. In ex vivo testing using pork knuckle, the gold- and carbon-based sensing patches exhibited comparable performance. The carbon-based sensing patch could detect 2 mL of extravasated fluid with an average resistance change of 51%. Thus, carbon is a potential alternative sensing material for extravasation detection because of its excellent performance, high-throughput manufacturing process, and cost-effectiveness. In future studies, the performance of carbon-based sensing patches can be validated in an actual clinical trial setting with patients.

## Figures and Tables

**Table 1:** Details of the volunteers for human trials.

Human Trial No.	Race	Age	Sex
V01	Chinese	37	Male
V02	Chinese	35	Male
V03	Malay	34	Male
V04	Malay	36	Male
V05	Indian	49	Male
V06	Filipino	36	Male
V07	Indian	54	Male
V08	Malay	27	Male
V09	Malay	36	Male

## Data Availability

Not applicable.
